# Fate of *Trypanosoma cruzi*, the causative agent of Chagas disease, in bed bugs after oral ingestion or intrathoracic injection

**DOI:** 10.1371/journal.pntd.0013568

**Published:** 2025-11-14

**Authors:** Sanam Meraj, Phillip Phung, Regine Gries, Carl Lowenberger, Gerhard Gries

**Affiliations:** Department of Biological Sciences, Simon Fraser University, Burnaby, British Columbia, Canada; University of Florida College of Medicine, UNITED STATES OF AMERICA

## Abstract

The parasite *Trypanosoma cruzi*, transmitted by kissing bugs, causes Chagas disease in millions of people in Central and South America. Here we studied potential transmission of *T. cruzi* by common bed bugs, *Cimex lectularius*. *Trypanosoma cruzi* populations in bed bugs were monitored after oral ingestion or intrathoracic injection, with parasite presence in tissues and excreta analyzed microscopically. Natural feeding and defecation behaviors were also observed. After bed bugs ingested *T. cruzi*-infected blood, the *T. cruzi* population in their anterior midgut steadily declined during days 1–7 but increased in their posterior midgut/hindgut and in their feces during days 4–7 and 7–10, respectively. No live or dead *T. cruzi* were observed in the bed bugs’ proboscis, salivary glands, and hemolymph, suggesting they did not breach the midgut. When bed bugs were injected intrathoracically with *T. cruzi* at a low concentration (10^3^ parasites/mL), no parasites were found in the hemocoel, but some were found up to one week after injection with a higher concentration (10^6^ parasites/mL). As *T. cruzi* was absent from the salivary glands of bed bugs, oral transmission during feeding is highly unlikely. As bed bugs did not defecate while feeding and invariably defecated away from their feeding site, transmission of *T. cruzi* via bed bug feces – in a mode similar to kissing bugs – is also unlikely. Human consumption of food contaminated with infected bed bugs is a possible, but untested, mode of transmission.

## Author summary

Blood-feeding triatomine kissing bugs transmit the parasite *Trypanosoma cruzi* which causes Chagas disease in millions of people in Central and South America. Here, we investigated whether blood-feeding common bed bugs, *Cimex lectularius*, also have the potential to transmit *T. cruzi*. When bed bugs ingested *T. cruzi*-infected blood, or when they were injected with *T. cruzi,* the parasites failed to enter the bed bugs’ salivary glands which is necessary for oral transmission. The presence of *T. cruzi* in the alimentary tract and feces of bed bugs supports a potential feces-related transmission mode through bed bug bite wounds inflicted on hosts or through otherwise broken host skin. However, because no bed bug defecated during natural feeding on a human arm, and all bed bugs moved away from their feeding site before releasing their first fecal droplet, the feces-related transmission mode seems unlikely. Once confirmed in future studies, these findings may help explain why there are no reports of bed bugs transmitting *T. cruzi* in natural settings.

## Introduction

*Trypanosoma cruzi*, the causative agent of Chagas disease, exemplifies the public health concerns posed by vector-borne parasites. These parasites are particularly prevalent in impoverished communities where they cause devastating health, social, and economic consequences [1–3]. Listed as one the world’s most neglected tropical diseases, Chagas disease predominantly affects Latin Americans, with an estimated 7 million people infected, leading to around 10,000 deaths yearly [4,5]. Typically transmitted via hematophagous triatomine kissing bugs, *T. cruzi* can also spread through blood transfusions, organ transplants, maternal transfer, and the ingestion of contaminated food, highlighting the complex transmission pathways of this parasite [6].

**Fig 1:**
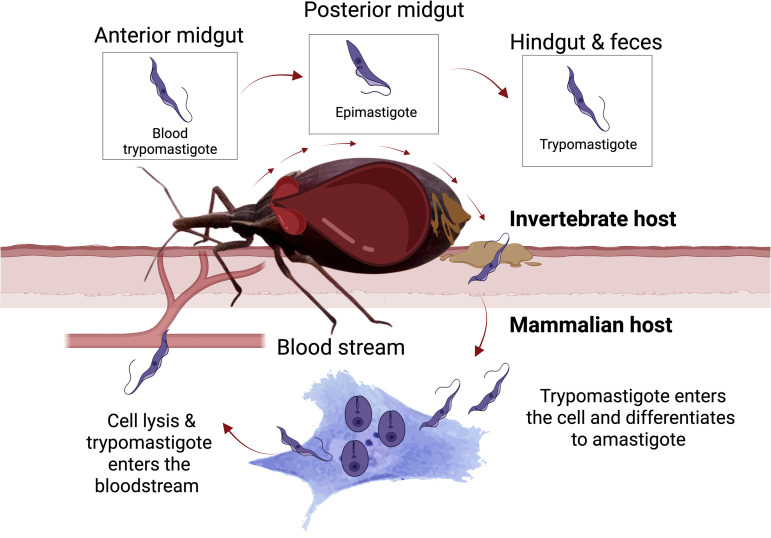
Life cycle and differentiation of *Trypanosoma cruzi* within kissing bug vectors (Triatominae) and mammalian hosts. In the anterior midgut of kissing bugs, *T. cruzi* trypomastigotes transition into amastigotes, which further differentiate into epimastigotes in the posterior midgut. Mature metacyclic trypomastigotes capable of infecting mammalian hosts are excreted with feces. In mammalian hosts, trypomastigotes enter the bloodstream, invade host cells, differentiate into amastigotes, and multiply intracellularly. This cycle illustrates the insect vector-mammalian host interactions critical to *T. cruzi* transmission and Chagas disease pathogenesis. Note: Modes of *T. cruzi* transmission other than those illustrated in this figure are also conceivable; see introduction/discussion for details. Graphs were created with GraphPad Prism version 10 (GraphPad Software, San Diego, CA, USA), and illustration components with BioRender.com. Additional artwork was drawn by SM.

Kissing bugs (Hemiptera: Reduviidae: Triatominae) are the primary vectors of *T. cruzi.* Kissing bugs ingest the trypomastigote form of *T. cruzi* together with the blood of infected vertebrate hosts (Fig 1). In the anterior midgut of kissing bugs, trypomastigotes differentiate into amastigotes, spheromastigotes, and epimastigotes. These forms attach to the posterior midgut wall, multiply, and then pass to the hindgut, where they attach to its cuticular lining and transform into metacyclic trypomastigotes. During or after feeding, the insect voids the infectious trypomastigotes with its feces and urine which can contaminate bite wounds inflicted on the host [7–10]. *Trypanosoma cruzi* enters hosts through skin lesions or mucous membranes [7,8]. Trypomastigotes, upon entering the mammalian bloodstream, invade host cells, differentiate into amastigotes, and multiply intracellularly. Strains of *T. cruzi* are currently grouped into six distinct typing units (DTUs; TcI to TcVI) [11,12], which are transmitted, or eliminated, depending on the insect host [13].

Common bed bugs, *Cimex lectularius* L. (Hemiptera: Cimicidae), are hematophagous insects that have been investigated as potential *T. cruzi* vectors [14–16], particularly in light of their global resurgence since the 1990s [17–20]. Although bed bugs harbour over 40 potential disease-causing pathogens, including bacteria, viruses, and parasites [4,17,19,21–24], there is no evidence that they transmit these pathogens to humans *in vivo*. Bed bugs acquired *T. cruzi* when they were fed experimentally on *T. cruzi*–infected mice, and subsequently transmitted *T. cruzi* to uninfected mice [15,25,26]. However, there are no reports that bed bugs transmit *T. cruzi* in natural settings, prompting the search for possible explanations. The potential explanation that *T. cruzi* infections reduce the bed bugs’ survival or reproduction was investigated but the data did not support this concept [26]. An alternative explanation for why bed bugs do not transmit *T. cruzi* in natural settings may lie in the bed bugs’ natural host feeding and defecation behaviour which may limit the potential for *T. cruzi* transmission but these behaviors have not yet been studied. That *T. cruzi* can be found in bed bugs for more than 320 days post-infection, can persist in the midgut throughout the bed bug moulting stages, and can be found in bed bug feces, all emphasize the need to further investigate the bed bugs’ vectorial potential [14,15].

Here we studied the fate and development of *T. cruzi* in bed bugs with the aim of determining potential transmission modes to vertebrates. Specifically, we investigated the bed bugs’ natural host feeding and defecation behavior, and the spatial and temporal distribution of *T. cruzi* in bed bug tissues and excreta after being ingested by, or injected into, bed bugs.

## Methods

### Laboratory rearing of bed bugs

The start-up colony of bed bugs was supplied by Harold Harlan (Crownsville, MD, USA) and supplemented with specimens collected from infested apartments in Vancouver (BC, Canada). Bed bugs were maintained as described previously [27]. Briefly, colonies were kept in the insectary of Simon Fraser University at a temperature of ∼24 °C, ambient relative humidity, and a photoperiod of 14 h light to 10 h dark. Groups of 150 bed bugs were maintained in 50-mL glass jars fitted with a square of cardboard (2 cm × 2 cm) at the bottom and a strip of cardboard (2 cm × 4 cm) diagonally across the jar as walk-on substrates. Bed bug groups were fed separately on the forearm of a volunteer (Regine Gries) once every month. For feeding, jars were covered with fine mesh, then inverted and pressed against the forearm so that the bed bugs could feed through the mesh. Under the guidelines of Simon Fraser University, no Institutional Review Board (IRB) approval was required for blood feeding bed bugs by Regine Gries (a study coauthor) who provided informed consent for this procedure.

### Origin of the *T. cruzi* isolate

The *T. cruzi* Y strain (ATCC Chagas 50832™, previously isolated from a Chagas disease patient in Brazil; obtained from Cedarlane, Burlington, ON, CA) was cultured in liver infusion tryptose (LIT) medium at 25 °C. For experiments, the density of parasites in mid-logarithmic growth phase was adjusted to 1 × 10^7^ parasites/mL, using a Neubauer chamber in an optical microscope for quantification.

### Infecting bed bugs with *T. cruzi* through oral ingestion and intrathoracic injection

Adult male bed bugs, blood-deprived for >20 days, were infected with *T. cruzi* through oral ingestion or intrathoracic injection. Whereas intrathoracic injection is not a natural mode of *T. cruzi* transmission, it was included here because we did not know whether *T. cruzi* can breach the gut barrier to the hemolymph, and we wanted to investigate the fate of *T. cruzi* when challenged with the immune responses in the hemocoel of bed bugs. For each timepoint (days 0, 1, 4, 7, 10, 20, and 30) in both the feeding treatment and the injection treatment, 10 individual bed bugs were separately dissected, each representing a functional replicate. Drawing on results of pilot studies, this timeline allowed detailed documentation of the parasites’ initial survival and subsequent proliferation and migration within their host. Data collections were repeated at least 3 times with new batches of parasites and bed bugs (200–300).

For oral ingestion of *T. cruzi,* bed bugs (200–300) in the treatment group were fed on defibrinated rabbit blood presented in a water-jacketed membrane feeder (Thermo Fisher Scientific Isotemp 2150 B14, USA) set to 37 °C, with stretched-out parafilm as the membrane. Rabbit blood was chosen because rabbits are one of many bed bug blood hosts and because it allows normal feeding, development, and reproduction of bed bugs. The blood was infected with 10^7^ parasites (epimastigotes)/mL in their log growth phase which had been cultured for 3–4 days in LIT medium, with cell density monitored daily using a hemocytometer. This seemingly high concentration of parasites (10^7^ parasites/mL; acute phase of parasitemia: 10⁴–10⁶ parasites/mL) was chosen to ensure that we would not underestimate potential survival of parasites in bed bugs and their potential transmission by bed bugs to new hosts. Bed bugs (200–300) in the control group fed on sterile rabbit blood which was also presented in a membrane feeder. Fully engorged bed bugs (150–200) in each group were isolated and kept in 50-mL glass jars (fitted with cardboard as walk-on substrate) to monitor for the presence and abundance of parasites immediately after their ingestion by bed bugs, and subsequently on days 1, 4, 7, 10, 20, and 30 post feeding.

For intrathoracic injection of *T. cruzi,* bed bugs (≥ 200) in the treatment group were injected with a 0.5-μL phosphate-buffered saline (PBS) solution containing either 10^3^ or 10^6^ parasites/mL, as determined by a hemocytometer. Bed bugs (≥ 200) in the control group were injected with 0.5-μL PBS. To capture the early phase of infection and to track the bed bugs’ immune responses, bed bug hemolymph was collected immediately after intrathoracic injection (day 0) and subsequently on days 1, 4, and 7.

### Quantitative presence of live (motile) *T. cruzi* in bed bugs after ingestion or injection

The presence and abundance of live (= motile) parasites were tracked immediately after their ingestion by bed bugs (upon feeding cessation), and subsequently on days 1, 4, 7, 10, 20, and 30. At each timepoint, 10 individual bed bugs (each representing a functional replicate) were separately dissected to determine the presence of live *T. cruzi* in the hemolymph, anterior midgut (S1 Fig), combined posterior midgut and hindgut, salivary glands, and feces. In all experiments, motile parasites were deemed alive, whereas immotile parasites – also stained with trypan blue – were deemed dead [14,16,28].

Bed bugs werepan surface-sterilized using 70% ethanol followed by two PBS washes to eliminate trace ethanol. Subsequently, each bed bug was dissected under a Carl Zeiss Stemi 2000-C stereoscopic microscope to isolate the midgut, salivary glands, hemolymph, and feces. To avoid any potential cross-contamination, midgut samples, in particular, were triple-washed with PBS. The midgut was divided into the anterior midgut and the posterior midgut/hindgut [29]. The midgut segments were then placed into tubes containing 50 μL PBS for homogenization and centrifugation. Live parasites in the supernatant of these samples were quantified using a hemocytometer.

Salivary glands of bed bugs were placed on a glass slide and ruptured using dissection scissors. The presence of live (motile) parasites within salivary glands was observed at magnifications of 400× and 1000× under a phase-contrast oil immersion microscope.

To extract hemolymph, a bed bug’s last abdominal segment was removed, and the abdomen was gently compressed for approximately 10 s to induce hemolymph flow, 0.5 μL of which was collected in an Eppendorf micro-tube, pre-lined with PBS to achieve a 50-fold dilution. Both diluted and undiluted samples were screened for the presence of live *T. cruzi* under a light microscope using phase contrast settings.

Feces from live individual bed bugs (n = 10) was obtained by exerting pressure on their abdomen. Excreted feces (~ 0.5 μL per bed bug) was diluted in PBS (50 μL) and then used to determine the presence of live *T. cruzi* with a light microscope equipped for phase contrast viewing.

Following injection of *T. cruzi* into bed bugs, we analyzed parasite presence in the bed bugs’ hemolymph, midgut, and salivary glands using the same procedures as described for parasite ingestion. To detect intermediary forms of the parasites, they were fixed in methanol and subsequently stained with Giemsa.

### Re-feeding experiments

To determine whether bed bugs infected with *T. cruzi* could transmit live *T. cruzi* during feeding, a re-feeding experiment was run. Bed bugs that had been fed on blood containing live *T. cruzi* were offered a second feeding opportunity 1, 4, 7, 10, 20, and 30 days later on non-infected blood. At each time point, 20 bed bugs were allowed to singly re-feed on sterile blood (1 mL) presented in a small membrane feeder. Once the bed bugs were fully engorged, the blood remaining in the artificial feeder was observed under the microscope for the presence of live or dead parasites to determine potential parasite transmission from salivary glands. Moreover, the salivary glands, midgut, and hemolymph of these 20 re-fed bed bugs were investigated for the presence of *T. cruzi*.

### Bed bug feeding and defecation behaviour

To determine the onset of defecation by bed bugs during or after blood feeding, 16 bed bugs that had been blood-deprived for 26 days were allowed to feed on the unclothed forearm of a volunteer (Regine Gries) at room temperature. To this end, bed bugs in groups of 4 were placed on the arm, and their feeding behaviour, duration of feeding (time between mouthpart insertion and feeding cessation), and the time elapsed between feeding cessation and first defecation events were recorded. All bed bugs could freely exit the feeding site on their own accord.

### Statistical analysis

Numbers of parasites in control and infected bed bugs were analyzed using the Kruskal-Wallis test. Control bed bugs were not expected to contain parasites but served as a reference for infected bed bugs in statistical analyses. The non-parametric Kruskal-test was used because data were not normally distributed and/or lacked heterogeneity of variance. Analyses were run using GraphPad Prism 8 software.

## Results

### Presence of live *T. cruzi* in bed bug tissues after ingestion of *T. cruzi*-infected blood

**Fig 2:**
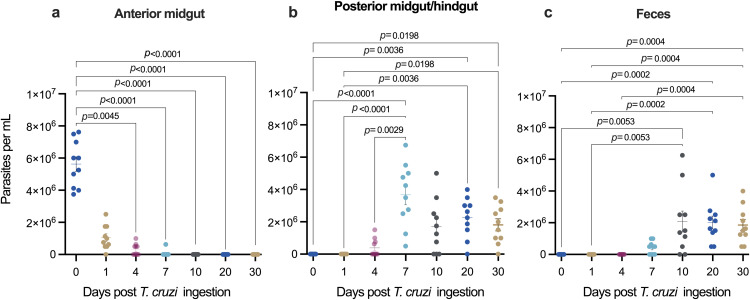
Spatial and temporal distribution of live *Trypanosoma cruzi* (Y strain) in bed bugs after ingestion of blood infected with live *T. cruzi.* Load of live *T. cruzi* parasites within the anterior midgut (a), posterior midgut/hindgut (b), and feces (c) immediately after feeding (day 0) or 1 to 30 days after oral ingestion. No parasites were observed in the proboscis, salivary glands and hemolymph at any time post-infection. The mean and standard error bars represent data of 10 replicates, with each replicate representing one bed bug (Kruskal-Wallis tests; data with p < 0.05 were considered statistically significant). Note: Very similar results were obtained with two additional cohorts of bed bugs (S1 Data).

**Fig 3:**
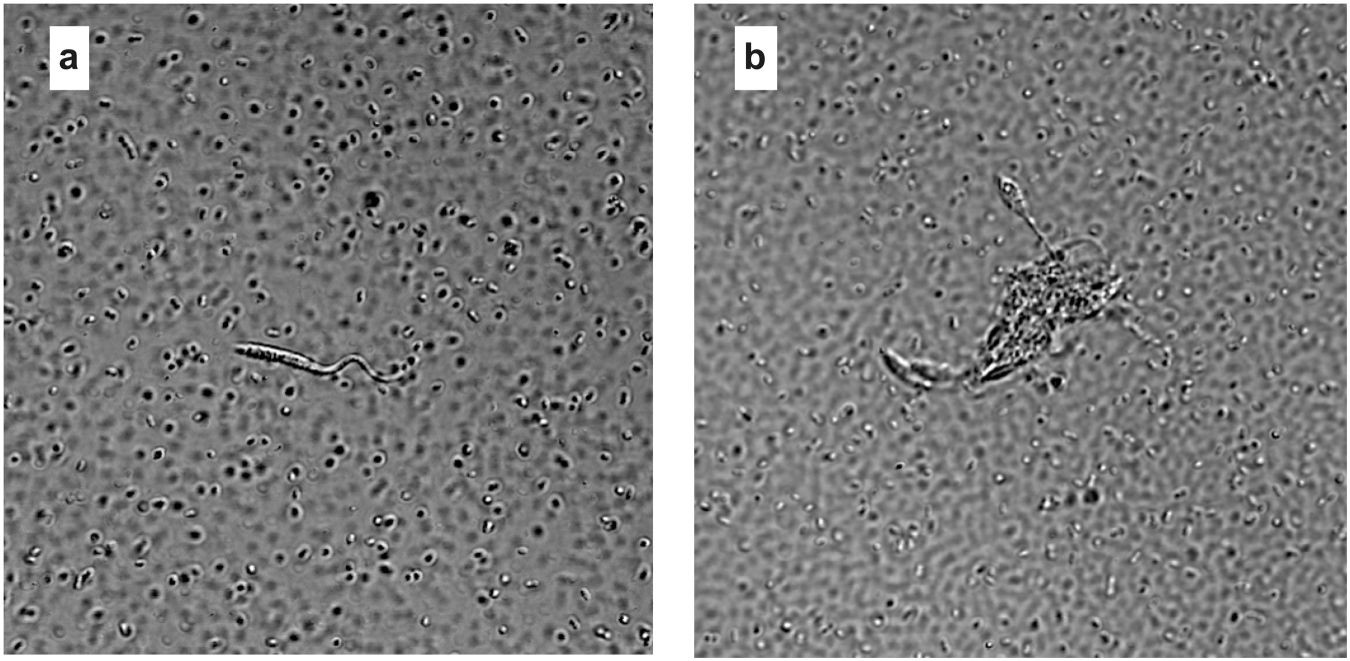
Flagellated forms of *Trypanosoma cruzi* in the posterior midgut/hindgut of bed bugs after ingestion of blood infected with live *T. cruzi.* (a) Single live *T. cruzi* parasite in a bed bug’s posterior midgut/hindgut. (b) Cluster of live *T. cruzi* parasites in a bed bug’s posterior midgut/hindgut. Panels a and b show different stages of the parasite. Dots illustrate gut microbiota. Photographs were taken by SM.

After bed bugs had ingested blood infected with live *T. cruzi*, live parasites were present in the anterior midgut of bed bugs on days 1, 4, and 7 post ingestion (pi), with live *T. cruzi* being notably absent on days 10 and >20 pi (see Fig 2a for parasite counts). During days 1–7 pi, counts of live *T. cruzi* parasites in the anterior midgut steadily declined. In contrast, in the posterior midgut and hindgut, no live parasites were present on day 1 pi but counts of live *T. cruzi* significantly increased during days 4–7 pi (Fig 2b), indicating that *T. cruzi* multiplied there, or moved from the anterior midgut to the posterior midgut/hindgut. Various developmental stages of *T. cruzi* were present (unquantified) in the bed bugs’ posterior midgut but were absent from the bed bugs’ feces. In excreted feces, live *T. cruzi* were first detected on day 7 pi, and on every timepoint thereafter (Fig 2c). At all timepoints, live *T. cruzi* were absent in the bed bugs’ proboscis, salivary glands, and hemolymph, but live *T. cruzi* were found in the bed bugs’ posterior midgut/hindgut (Figs 2 and 3). These findings describe both temporal and spatial colonization dynamics of *T. cruzi* within bed bugs and identify potential windows of transmission risks and modes.

### Survival of *T. cruzi* in the bed bugs’ hemolymph after intrathoracic injection

When bed bugs were injected with live *T. cruzi* at 10^6^ parasites per mL, all bed bugs died within 7 days, and some bed bugs contained live *T. cruzi* (Table 1). When bed bugs were injected with live *T. cruzi* at 10^3^ parasites per mL, all bed bugs survived more than 7 days, and no live *T. cruzi* were found in the bed bugs’ hemolymph (Table 1), or midgut, at any timepoint. All bed bugs injected with a PBS control survived.

**Table 1:** Survival of *Trypanosoma cruzi* in the bed bugs’ hemolymph over 7 days after intrathoracic injection of *T. cruzi* at 10^3^ or 10^6^ parasites per mL.

Days post injection	10^6^ Parasites/mL		10^3^ Parasites/mL	
	Live	Dead	Live	Dead
1	(5/10)	(10/10)	(0/10)	(10/10)
4	(2/10)	(10/10)	(0/10)	(10/10)
7	(2/10)	(10/10)	(0/10)	(2/10)

Data represent the number of bed bugs (out of 10) that contained live (motile) or dead (immotile) parasites; dead parasites also stained with trypan blue.

### *Trypanosoma cruzi* parasites persist in bed bugs but are not orally transmitted by bed bugs during feeding

Bed bugs that had ingested *T. cruzi-*infected blood (10^6^ live parasites per mL) during a first blood meal – and then fed subsequently on sterile blood 2, 4, 7, 10, 20, and 30 days later – did not “transmit” *T. cruzi* to the sterile blood during their second feeding. Whereas live *T. cruzi* were present in the midgut of these bed bugs, they were not able to migrate from the midgut to establish in the salivary glands. Apparently, *T. cruzi* can persist in bed bugs but is not transmitted during feeding under the conditions tested here.

### Bed bug feeding and defecation behaviour

The 16 bed bugs we recorded fed – on average – for 536 s (range: 369–807 s; median = 494.4 s) on the forearm of the volunteer. No bed bugs defecated while feeding. After bed bugs had completed feeding, an average of 79 s (range: 3.0–351.0 s; median = 51.6 s) elapsed before bed bugs excreted a single fecal droplet onto the arm, invariably away from their feeding site. Engorged bed bugs moved off the arm.

## Discussion

After bed bugs ingested parasites, the population of live *T. cruzi* in their anterior midgut steadily declined during days 1–7 post ingestion, with no live *T. cruzi* being detectable past day 7. Conversely, after day 7, the population of live *T. cruzi* in the posterior midgut/hindgut of bed bugs and in their feces increased, indicating parasite multiplication or migration from the anterior gut region (Fig 2). Bed bugs did not “transmit” live or dead *T. cruzi* parasites via feeding, but the presence of live *T. cruzi* in bed bug feces supports potential transmission routes, whereby *T. cruzi* parasites exit the feces and enter bite wounds, or *T. cruzi-*infected bed bugs contaminate food and are ingested by humans. However, although bed bug feces contained infective trypomastigotes, we do not know whether these motile parasites were viable and able to enter a vertebrate and infect mammalian cells. Various studies demonstrated successful infection of naïve animals with *T. cruzi* from bed bug feces [14–16,25] but whether these infections were successful due to the specific *T. cruzi* strains tested or due to specific experimental protocols is not clear. Following intrathoracic injection of live *T. cruzi,* live parasites persisted in bed bugs for up to 7 days.

**Fig 4:**
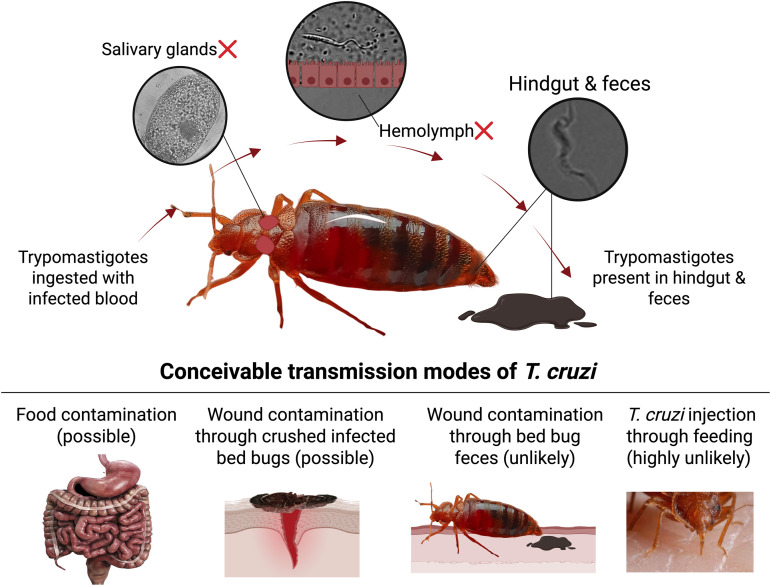
Life cycle of *Trypanosoma cruzi* in bed bugs and evidence for very low risk of *T. cruzi* transmission via bed bug feeding and fecal contamination of bite wounds. During days 1–7 post-ingestion (pi) of *T. cruzi*-infected blood by bed bugs, *T. cruzi* counts in the bed bugs’ anterior midgut steadily declined (Fig 2a), suggesting that the bed bugs’ immune response eliminated the parasites. No live *T. cruzi* parasites were present in the posterior midgut and hindgut on day 1 pi (Fig 2b), but during days 4–7 pi, *T. cruzi* counts in the posterior midgut and hindgut significantly increased (Fig 2), indicating multiplication of *T. cruzi*. As *T. cruzi* parasites were first detected in excreted fecal matter on day 4 pi (Table 2), and bed bugs did not defecate while feeding, and invariably defecated away from their feeding site, transmission of *T. cruzi* via bed bug feces is not very likely. As *T. cruzi* parasites were absent at all times from the bed bugs’ proboscis, salivary glands, and hemolymph (as illustrated in this figure), *T. cruzi* transmission through bed bug feeding on human hosts is highly unlikely. Graphs were created with GraphPad Prism version 10 (GraphPad Software, San Diego, CA, USA), and illustration components with BioRender.com. Additional artwork was drawn and the photographs were taken by SM and Dr. Adam Blake.

That the *T. cruzi* population rapidly declined in the bed bugs’ anterior midgut suggests a lack of suitable binding receptors [30,31] or unfavourable conditions for survival and proliferation in this section of the bed bugs’ alimentary canal. Rapidly declining populations of ingested parasites in the anterior midgut is also common in the kissing bug *Rhodnius prolixus* and in the tsetse fly *Glossina morsitans,* after ingestion of *T. cruzi* and *T. brucei,* respectively [32,33]. The combined data indicate that parasite numbers fall rapidly in the anterior midgut. Alternatively, parasites may migrate from the anterior to the posterior midgut. This latter explanation, however, is not supported in our study. The parasite load in the anterior midgut declined during days 0–1 post infection, but no parasites were concurrently detected in the posterior midgut (Figs 2a,b and 4). Our data support the hypothesis by Ferreira et al. [33] that a significant proportion of parasites is eliminated in the anterior midgut of the host insect shortly after ingestion, likely due to significant changes in temperature and pH [33]. Between days 4 and 7 post infection, the *T. cruzi* population increased and remained stable in the bed bugs’ posterior midgut, indicating that the posterior midgut and hindgut are pivotal sites for parasite proliferation and differentiation (Figs 2 and 4).

**Table 2:** Assessment of potential *Trypanosoma cruzi* transmission by infected bed bugs when feeding on sterile blood on days 1–30 post initial *T. cruzi* infection.

Days of re-feeding	Presence of live *T. cruzi* parasites			
	Fed-on blood	Salivary gland	Whole midgut	Feces
1	(0/10)	0/10	(10/10)	(0/10)
4	(0/10)	0/10	(10/10)	(1/10)
7	(0/10)	0/10	(10/10)	(8/10)
10	(0/10)	0/10	(10/10)	(10/10)
20	(0/10)	0/10	(10/10)	(10/10)
30	(0/10)	0/10	(10/10)	(10/10)

Note that all 10 *T. cruzi*-infected bed bugs contained live parasites in their midgut at all timepoints after ingestion and that only one bed bug excreted live parasites with its feces by day 4 after *T. cruzi* ingestion. Out of 10 bed bugs, none contained live or dead *T. cruzi* in their salivary glands, and none transmitted live or dead *T. cruzi* to sterile blood during re-feeding.

The transient presence of *T. cruzi* in the bed bugs’ anterior midgut, and their population increase and sustained abundance in the bed bugs’ posterior midgut and hindgut, resemble the colonization dynamics of *T. cruzi* in host kissing bugs [7,8,34,35].

Some distinct intermediary forms of *T. cruzi* were present in the bed bugs’ posterior midgut but were absent from bed bug feces. These observations refine our understanding of the *T. cruzi* differentiation process within insect hosts, suggesting that the posterior midgut of host insects plays a vital role in parasite differentiation. *In vitro* studies – that analyzed the differentiation of cultured cell-derived trypomastigotes to epimastigotes – revealed that epimastigogenesis occurs by conversion of intermediary forms which, in turn, give rise to epimastigotes [36,37]. Results of our *in vivo* experiments suggest a similar route of differentiation, including the occurrence of non-replicative amastigote-like intermediary forms (Fig 3).

Feces samples of *T. cruzi-*infected bed bugs contained trypomastigotes as early as day 4 post infection. These data closely align with the reported life cycle of *T. cruzi* in kissing bugs [7,8,34,35,38–40], where trypomastigotes in the anterior midgut differentiate into intermediary stages, which then multiply in the posterior midgut and finally move to the hindgut to be excreted together with feces. The presence of live *T. cruzi* in the bed bugs’ midgut and feces supports a potential, but unlikely, feces-related transmission mode similar to that in kissing bugs. As *T. cruzi* was absent from the salivary glands of bed bugs, salivary transmission of parasites during feeding is highly unlikely. In kissing bugs – as in bed bugs – *T. cruzi* cannot breach the perimicrovillar membrane of the midgut [34,35,38–40], and cannot enter the hemocoel and ultimately the salivary glands. Furthermore, human consumption of food contaminated with *T. cruzi-*infected kissing bugs (‘food-borne transmission’) presents another transmission mode, contributing to outbreaks of Chagas disease in the Americas [41,42]. This food-borne mode of ‘Chagas disease transmission’ could also apply to, but seems unlikely for, *T. cruzi*-infected bed bugs.

### Survival of *T. cruzi* strains in bed bugs and kissing bugs

After bed bugs had ingested live *T. cruzi,* live parasites were present in the midgut and feces, but no live or dead parasites were present in the bed bugs’ hemolymph at any time, indicating that *T. cruzi* failed to enter the hemocoel. In our study, all bed bugs injected with live 10^6^ parasites/mL died within a week of injection. Live parasites were observed in the hemolymph of live bed bugs for up to one week. Bed bugs injected with live *T. cruzi* at 10^3^ parasites/mL lived longer than one week, with no live parasites found in the hemolymph or other tissues, and only dead parasites were present in the hemolymph across all timepoints. All results together illustrate that the Y strain of *T. cruzi* cannot breach the gut barrier to the hemolymph but can survive in the hemolymph when injected into the hemocoel at high concentrations.

Kissing bugs deploy a layered defense against *T. cruzi* and other parasites and pathogens. Immune responses by *Triatoma* spp. and *Rhodnius* spp. to *T. cruzi* infections include physical barriers, humoral and cellular responses (e.g., lysozyme and prophenoloxidase activity), parasite agglutination, and activation of specific antimicrobial peptides (AMPs) such as prolixicins, defensins and lysozymes [35,43]. The microbiota of triatomines further affect various *T. cruzi* strains [44,45]. Moreover, *R. prolixus* demonstrates strain-specific clearance and agglutination of *T. cruzi* [35,43]. In contrast, most immune factors that bed bugs express in response to parasite infections have yet to be investigated.

In a previous study [46], we demonstrated that bed bugs express the AMP CL-prolixicin2 in their fat bodies, and that synthetic CL-prolixicin2 kills *T. cruzi* and impedes its proliferation. The collective data suggest that prolixicin expression in bed bugs may contribute to the elimination of *T. cruzi* (Y strain) from their hemocoel. Limited survival of *T. cruzi* at high concentrations in the hemolymph of bed bugs [39,47] warrants investigation into what extent infection levels determine the outcome of immune responses and the involvement and contributions of various immune factors.

### Bed bug defecation behaviour and possible modes of *T. cruzi* transmission by bed bugs

The distinctively different feeding and defecation behaviour of bed bugs and kissing bugs may contribute to the contrasting significance of these insects as parasite vectors. In our study, no bed bug defecated during feeding, and all bed bugs moved away from their feeding site before releasing their first fecal droplet, on average 79 s later. These results differ from those of previous reports that kissing bugs and bed bugs have a similar defecation index [16,48], the fraction of insects defecating within 10 min multiplied by the average number of defecations in 10 minutes [16,48]. These contrasting results are likely due to contrasting experimental designs. In our behavioural experiment, bed bugs fed naturally on the unclothed forearm of a volunteer, rather than through a membrane feeder, and they could freely exit the feeding site on their accord. The natural feeding and defecation behaviour observed in our experiment would reduce the risk of feces-related *T. cruzi* transmission, and thus may explain, in part, the epidemiological insignificance of bed bugs in the transmission of *T. cruzi*. Kissing bugs, in contrast, defecate both during and shortly after feeding, with most (70–90%) adult kissing bugs defecating on the host within one minute after the onset of feeding. This early onset of defecation in kissing bugs is crucial for the transmission of *T. cruzi* through broken skin when feces of an infected kissing bug is inadvertently rubbed into the bite wound [7,8,39,49,50].

There is a potential risk of food-borne transmission of *T. cruzi* by bed bugs similar to the outbreaks of Chagas disease related to the ingestion of food contaminated with *T. cruzi*-infected kissing bugs [4]. Accidental ingestion of food contaminated with *T. cruzi*-infected bed bugs, or their feces, could result in transmission. Transmission of *T. cruzi* might also occur if people touch their eyes or mouth with their hands soiled with *T. cruzi-*infected bed bug feces, or if an infected bed bug is accidentally crushed against human skin and the gut contents are released into a wound. Although bed bug bites cause itchiness [22,51,52], transmission of *T. cruzi* by rubbing it into itchy and broken skin is unlikely considering the typically delayed onset of such itchiness, and the bed bugs’ distinct feeding and delayed defecation behaviour.

### Public health implications of bed bugs in Chagas disease-endemic regions: A case for enhanced surveillance

Our findings have critical implications for public health, particularly in highly populated urban settings. People living in shelters or crowded dwellings with poor hygiene and large bed bug infestations might be at elevated risks of exposure to *T. cruzi*-infected bed bugs and potential *T. cruzi* infection. The effects of sympatric bed bug and kissing bug populations on *T. cruzi* transmission should be studied, particularly because the potential roles of bed bugs as vectors of the different *T. cruzi* typing units are poorly understood.

## Conclusion

Y strain *T. cruzi* trypomastigotes were not transmitted through bed bug bites but when ingested may persist in bed bugs. The parasites did not breach the perimicrovillar membrane of the bed bugs’ midgut, enter the hemocoel, and colonize the salivary glands, which is necessary for oral transmission. The presence of *T. cruzi* in bed bug feces supports a potential feces-related transmission mode. However, the bed bugs’ natural host-feeding and delayed defecation behaviour – which had not yet been studied in the context of potential *T. cruzi* transmission – would likely limit feces-related transmission. To support this tentative conclusion, the host-feeding and defecation behaviour of a larger bed bug cohort including females and nymphs should be studied. The potential risk of food-borne transmission of *T. cruzi* by bed bugs, similar to what has been reported with kissing bugs, should also be explored as a putative mode of transmission.

## Supporting information

S1 Fig Dissected gut of a recently blood-fed bed bug showing distinct anatomical regions. The anterior midgut is visibly engorged with fresh blood, while the posterior midgut and hindgut appear narrower and less distended.
(DOCX)

S1 Data Raw data and statistical analysis for Fig 2, Spatial and temporal distribution of live *Trypanosoma cruzi* (Y strain) in bed bugs after ingestion of blood infected with live *T. cruzi.* (XLSX)
