# Supplementary material for: Fate of Trypanosoma cruzi, the causative agent of Chagas disease, in bed bugs after oral ingestion or intrathoracic injection
Source: PLoS Negl Trop Dis. 2025 Nov 14;19(11):e0013568. doi: 10.1371/journal.pntd.0013568 (PMC12671806; doi:10.1371/journal.pntd.0013568)
Supplement: S1 Fig — The anterior midgut is visibly engorged with fresh blood, while the posterior midgut and hindgut appear narrower and less distended. (DOCX) [file pntd.0013568.s001.docx]

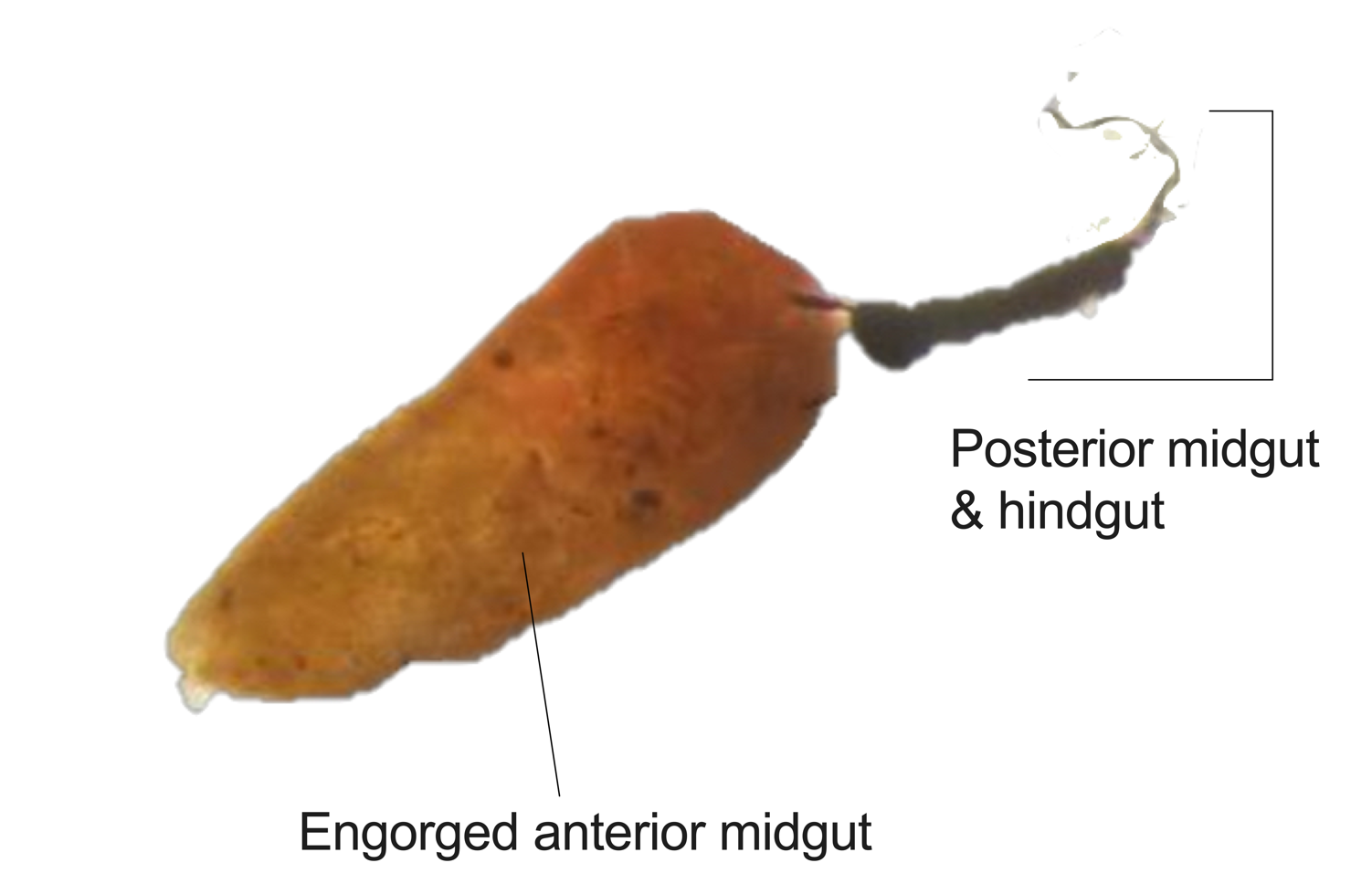


**S1 Fig.** Dissected gut of a recently blood-fed bed bug showing distinct anatomical regions. The anterior midgut is visibly engorged with fresh blood, while the posterior midgut and hindgut appear narrower and less distended.
